# Digital Image Correlation Characterization and Formability Analysis of Aluminum Alloy TWB during Forming

**DOI:** 10.3390/ma15155291

**Published:** 2022-07-31

**Authors:** Jie Wu, Yuri Hovanski, Michael Miles

**Affiliations:** 1 Department of Intelligent Manufacture and Mechanical Engineering, Hunan Institute of Technology, Hengyang 421002, China; 2 Department of Manufacturing Engineering, Brigham Young University, Provo, UT 84602, USA; yuri.hovanski@byu.edu (Y.H.); mmiles@byu.edu (M.M.)

**Keywords:** formability, limiting dome height, digital image correlation

## Abstract

The formability of aluminum alloy 5754-O tailor-welded blanks prepared by friction stir welding was studied experimentally. The strain evolution and deformation during limiting dome height experiments were studied using digital image correlation and the ARAMIS software. The influence of the sheet thickness of the base materials on the punch loading, fracture strain and formability were investigated experimentally. It was found that the punch loading, fracture strain and limiting dome height values increase with the increasing sheet thickness of the base materials. A linear relationship between the limiting dome height value and the sheet thickness was demonstrated. An increase of 16.8% in the fracture strain of aluminum tailor-welded blanks was observed for an increase of 36% in sheet thickness. This paper provides a methodology for experimentally determining the forming limits of aluminum alloy tailor-welded blanks accurately.

## 1. Introduction

Automotive lightweighting technology includes mass reduction via material selection and structural design [1,2,3]. Reducing weight through material selection requires design modifications with low-density materials, such as magnesium alloys, aluminum alloys, ultra-high-strength steels and high-performance plastics or composites to replace the typical steel alloys used in automotive structural design. Conversely, reducing weight with structural design requires mass optimization based on local performance requirements. Technologies that enable mass reduction with both material selection and structural design have become extremely competitive solutions for future automotive designs [1]. Tailor-welded blanks (TWBs) are one of the technologies that have been shown to enable mass reduction through both material selection and structural design [2,4]. Numerous studies show the application of TWBs has potential to reduce the weight of body-in-white structures with improved crashworthiness [1,2,3,4]. By moving from steel to aluminum TWBs, mass reduction is achieved through material selection, as the densities of aluminum alloys are lower than their steel counterparts. As TWBs can be produced of dissimilar alloys and dissimilar thicknesses, further weight reduction can be achieved for automotive applications based on structural design, which enables local design requirements to be matched with alloy and/or thickness. An example of such mass optimization was documented for a front-door inner panel, where 5 kg was reduced by using aluminum alloy tailor-welded blanks compared to steel tailor-welded blanks [5], and dissimilar thicknesses were used to enable local structural requirements at the door hinge without adding weight to the entire panel.

Understanding the forming behavior and the formability of TWBs is key to their practical application. Initial investigations of the formability of TWBs focused on understanding the variation in the forming limit diagram [6,7,8,9] resulting from both the reduction in the ductility of the weld metal and combinations of dissimilar thicknesses and alloys. Several studies specifically evaluated the influence that strength or thickness ratios have on the overall formability of the TWB [7,10]. Other studies focus on the influence of welding methods and parameters on the formability of TWBs [5,11,12,13], with recent studies working to improve friction stir welding (FSW) with ultra-sonic vibration [14]. Numerous studies focus on the optimization of FSW specifically for the formability of TWBs. These studies focus on tooling and weld parameters, with a consensus that increasing welding speed leads to improved formability [5,14,15]. Further evaluation of the influence of welding on the formability was reported by Parente et al. [11], showing the influence of weld direction on the overall formability of TWBs.

In parallel with significant evaluations of the effect of welding parameters related to the formability of TWBs, many researchers have focused on developing numerical tools to predict aluminum formability [16,17,18]. To further support the development of numerical models and characterization of formability, LDH testing has been used successfully by many researchers to measure the formability of aluminum TWBs [10,14,16]. One of the key issues of formability prediction is determining the strain distribution just before the fracture of the failed specimen [19]. While several visual tools have been used historically to measure local strain variations, digital image correlation (DIC) is becoming the standard [20,21,22,23].

In this paper, the forming behavior of aluminum TWBs is studied by using the LDH test with an integrated digital image correlation (DIC) system, ARAMIS (GOM, Braunschweig, Germany). The influence of the thickness of the base materials on the punch loading and formability were investigated. It was found that the punch loading and LDH values increase with the thickness of base materials. This paper provides a method for determining the formability of aluminum alloy TWB accurately.

## 2. Experimental Procedures

### 2.1. Base Materials and Welding Conditions

The material investigated in this study is a work-hardenable aluminum alloy, 5754-O, with original thicknesses equal to 2.2 mm, 2.7 mm and 3 mm. AA5754 has been an alloy of choice for the automotive community owing to its excellent ductility and formability characteristics [24]. The chemical composition and mechanical properties are shown in Table 1 and Table 2, respectively.

AA5754 is prone to spatter, porosity, thermal crack and other defects in the preparation of tailor-welded blanks by traditional laser welding [25]. In this study, FSW is used to fabricate the AA5754 tailor-welded blanks and its process parameters are as follows: the rotation speed is 1950 RPM, the welding speed is 3 m/min, and the inclination angle of the stirring pin is 1°. The FSW tool is made of H13 tool steel with a shoulder diameter of 12 mm and a probe length of 3.0 mm. The probe is tapered at 10° and has three flats at 120° from each other.

### 2.2. Limiting Dome Height Tests

**Figure 1:**
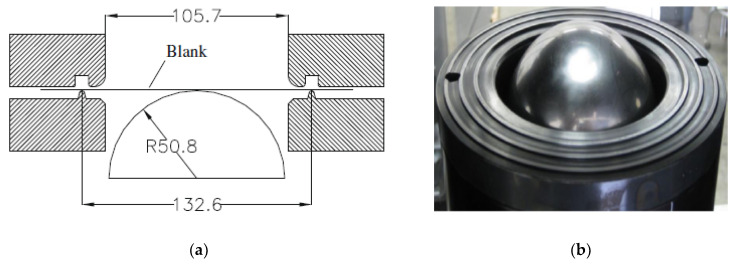
Schematic diagram (**a**) (Unit: mm) and punch and lower clamp of LDH test (**b**).

There are many methods to characterize the formability of TWBs. The most commonly used methods are Erichsen tests, forming limit curve tests [26] and LDH tests [27]. In the LDH tests, a hemispherical punch with a diameter of 101.6 mm was used for sheet metal stamping, as shown in Figure 1a. The corresponding forming height when sheet metal fractured was called the LDH value. The higher the LDH value, the better the formability of sheet metal. Figure 1b shows the punch and lower clamp of LDH experimental device and the physical drawing of the lower blank holder device. The upper blank holder device has a locking ring matching with the lower blank holder to ensure that the part of the blank holder is completely fixed in the forming process. A layer of lubricant is required on the surface of the sample before stamping.

**Figure 2:**
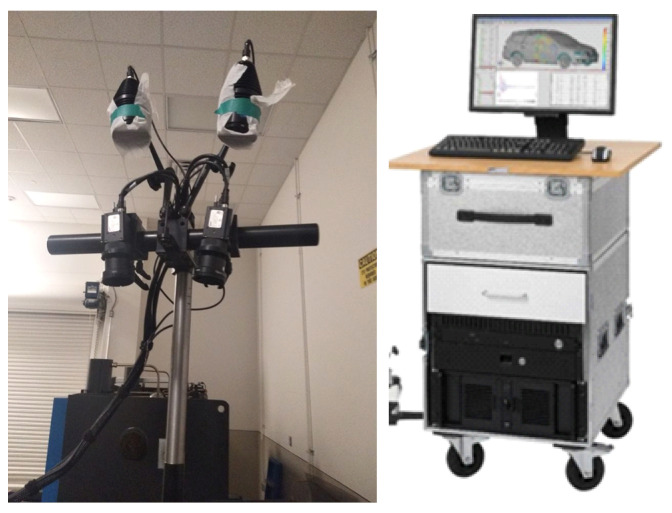
Camera and data processing system of ARAMIS.

**Figure 3:**
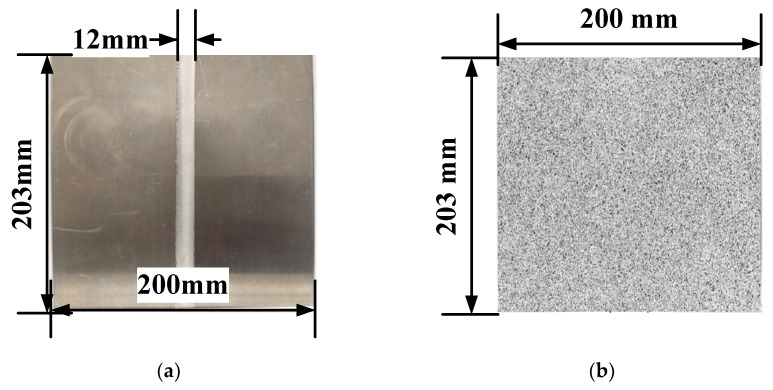
The morphology and size of AA5754 TWB (**a**) before spraying and (**b**) after spraying.

The ARAMIS digital image correlation (DIC) system (GOM, Braunschweig, Germany) was used to accurately record and measure the deformation and strain of the TWB during the LDH experiment, as shown in Figure 2. ARAMIS is a dynamic online strain-testing system based on the DIC method. Its advantage lies in that it can record the deformation of samples in the loading process as a function of time. The principle of strain measurement within the DIC system is as follows: Firstly, a square pixel area is selected in the sample, and the displacement vector of the pixel point is obtained by calculating the position changes of the pixel point before and after deformation, and the displacement field and strain field of the sample are obtained. In order to calculate the displacement field and strain field of the sample, random speckle is sprayed on the surface of the sample before testing. The process is as follows: (1) The sample surface is sprayed with alcohol to remove any stains or grease; (2) A layer of matte white paint is sprayed on the dry and clean sample surface; (3) A layer of speckles is sprayed using matte black paint on the dry surface of the white paint. Figure 3 shows the aluminum alloy tailor-welded blanks before and after speckle fabrication and their corresponding size.

## 3. Results and Discussion

### 3.1. Strain and Displacement Distribution and Evolution

**Figure 4:**
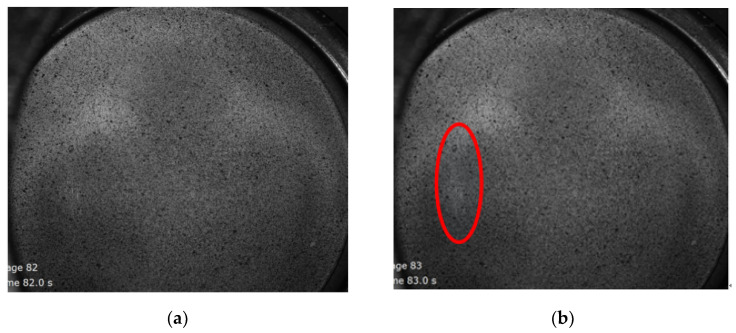
Surface of the deformed AA5754 tailor-welded blanks with thickness 3 mm: (**a**) prior to fracture (stage 82) and (**b**) after fracture (stage 83). The location of red circle is the fracture location.

**Figure 5:**
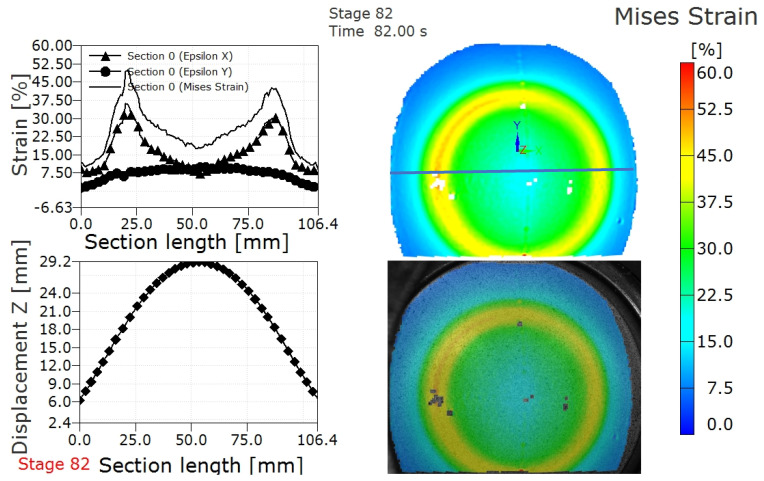
Strain distribution and displacement distribution along the section and Mises strain contour map in the deformed AA5754 TWBs with thicknesses of 3 mm prior to fracture.

The ARAMIS strain testing system can record the deformation, strain distribution and displacement distribution of formed parts during the LDH experiment in real time. The frequency at which the ARAMIS system gathers images during the forming process is 1 frame/second. Figure 4 shows the deformed TWBs prior to and after fracture in an LDH test. It can be seen from Figure 4 that the deformed parts are spherical in the deformation process, which is determined by the shape of punch, as shown in Figure 1b. Figure 5 shows the strain and displacement profile along a section perpendicular to the weld, marked with the horizontal blue line on the Mises strain plot in the upper right hand corner, prior to the fracture in the TWBs produced from the 3.0 mm thick sheet. The plots shown in Figure 5 report the formability of the TWB during strain localization just prior to fracture. As can also be seen from Figure 5, the strain distribution on LDH samples (prior to fracture) have two peaks symmetrically along the cross section. The same strain distribution is also obtained in this author’s earlier works [10], both in experimental and finite element simulations. Similar results were also found in steel tailor-welded blanks [7,17,19]. It can also be concluded that the major strain is much larger than the minor strain from the strain profile, as shown in Figure 5, where the epsilon X represents major strain while the epsilon Y represents minor strain. The displacement distribution (as shown in Figure 5) along the section could be considered as the deformed profile of the AA5754 TWBs shown in Figure 4a.

**Figure 6:**
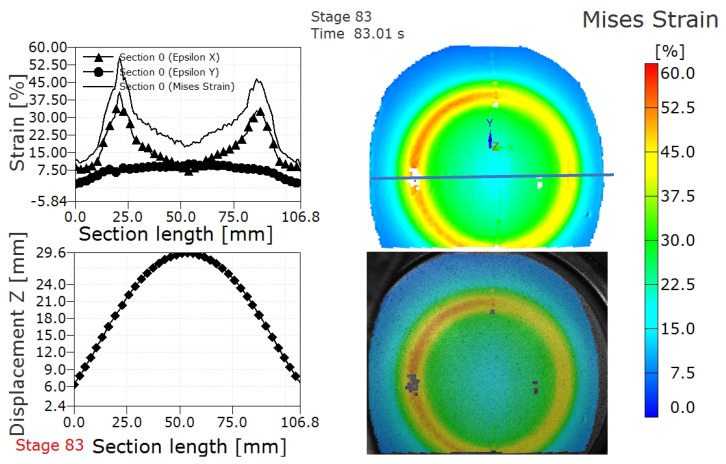
Strain distribution and displacement distribution after fracture of a 3 mm thick AA5754 TWB along the path (blue line) along with Mises strain and DIC overlay on the LDH specimen.

Figure 6 shows the strain and displacement profile along a section perpendicular to the weld after strain localization and fracture in the AA5754 TWBs produced with 3.0 mm thick sheet. Compared with the results obtained prior to fracture, as shown in Figure 5, the distribution profile is similar, while the value of strain is larger after fracture. The Mises strain at this stage (stage 82) can be considered the fracture strain in the AA5754 TWB produced with the 3.0 mm thick sheet, which shows the fracture strain is 48.5%, as shown in Figure 5 and Table 3. The strain evolution between strain at localization and strain at fracture can be extracted from comparison of Figure 5 and Figure 6.

**Figure 7:**
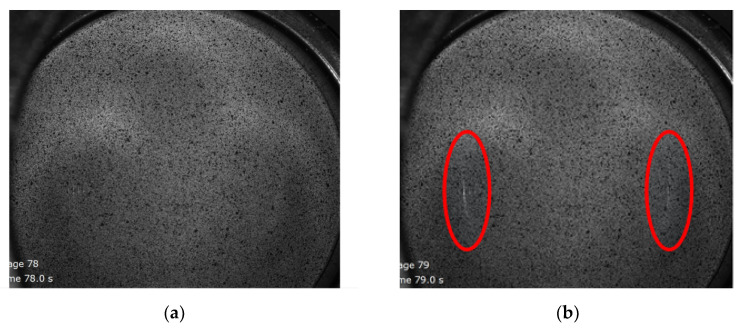
Surface of the deformed AA5754 tailor-welded blanks with thickness 2.2 mm: (**a**) prior to fracture (stage 78) and (**b**) after fracture (stage 79). The location of red circle is the fracture location.

**Figure 8:**
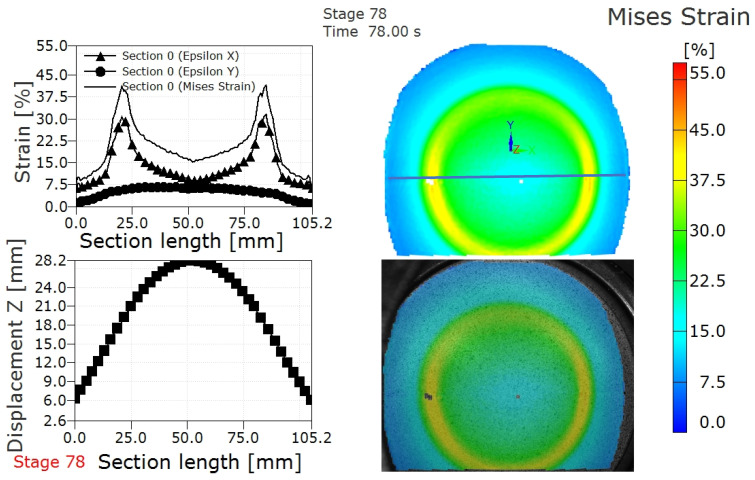
Strain distribution and displacement distribution along the section and Mises strain contour map in the deformed AA5754 tailor-welded blanks with thickness 2.2 mm prior to fracture.

**Figure 9:**
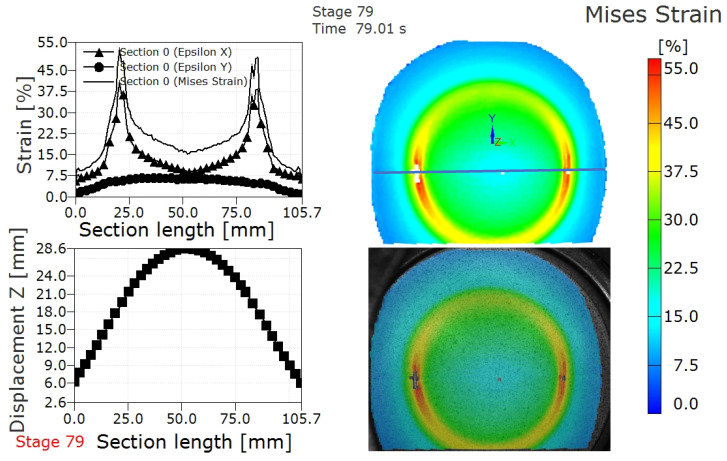
Strain distribution and displacement distribution along the path and Mises strain in the AA5754 tailor-welded blanks with thickness 2.2 mm after fracture.

Similar strain and displacement profiles to those presented for the 3 mm thick TWBs are presented for TWBs produced with a 2.2 mm thick sheet in Figure 7, Figure 8 and Figure 9. Figure 7 shows the deformed surface of AA5754 TWB produced with the 2.2 mm sheet prior to and after fracture. The deformation stages were stage 78 (prior to fracture) and stage 79 (after fracture). The strain distribution and displacement distribution along the section along with the Mises strain contour map of the deformed TWBs from the 2.2 mm sheet prior to and after fracture are shown in Figure 8 and Figure 9, respectively. The fracture strain of the 2.2 mm thick TWB was 41.5%, and the corresponding displacement (considered to be the LDH) was lower than that of the TWB produced with the 3.0 mm AA5754 sheet. The specific details that can be seen in Figure 8 and Figure 9 are summarized for comparison in Table 3. This comparison shows that increasing sheet thickness leads to an increase in the overall punch load, which is intuitive based on the increased bearing capacity of the thicker sheet. However, this comparison also shows that the increased thickness leads to higher LDH values commensurate with increased elongation in the thicker sheet, which suggests that material ductility alone does not limit the formability.

**Figure 10:**
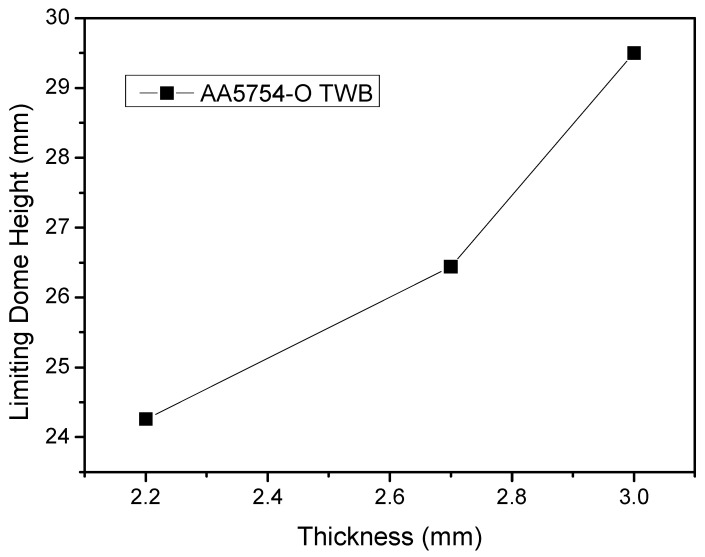
Relationship between the thicknesses of AA5754 tailor-welded blanks and the LDH.

Figure 10 shows the relationship between the thicknesses of AA5754 TWBs and the LDH values, as LDH values of 2.2 mm, 2.7 mm and 3.0 mm TWBs are reported. It can be concluded that the LDH values increase as the thickness of AA5754 TWBs increase, and that the relationship is nearly linear with material thickness. A linear trend line for Figure 10 would have an R^2^ value of 0.94.

### 3.2. Punch Loading—Punch Stroke Curve and Formability

**Figure 11:**
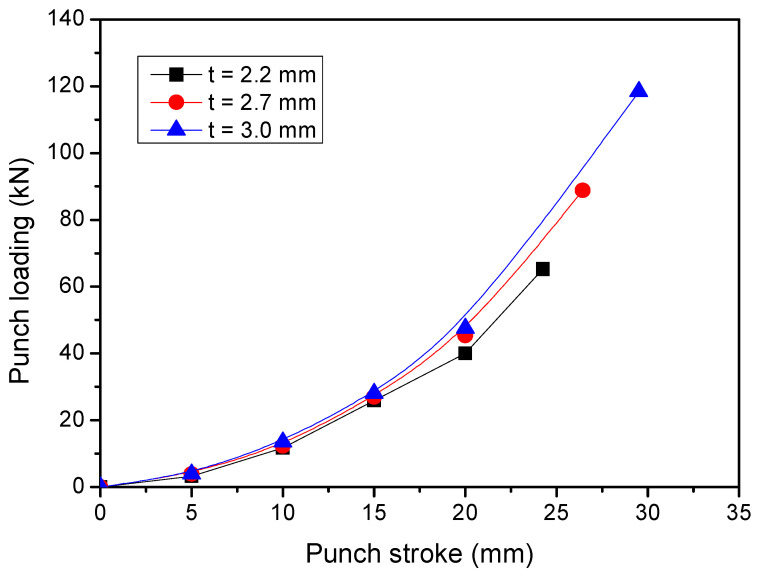
Punch stroke and punch loading curve of AA5754 tailor-welded blanks with different thickness.

The punch loading and punch stoke curve of the LDH press on the AA5754 TWBs with different thickness are compared in Figure 11. As expected, the punch loading required to deform the TWBs with the 3.0 mm thick sheet was higher than that required for the TWBs with the 2.2 mm and 2.7 mm sheets because more energy was required to deform the thicker material. The corresponding maximum punch stroke at peak loading was considered to be the LDH value of the TWBs examined herein. The LDH values, punch loading, maximum strain and deform stage of the TWBs with different thicknesses were summarized in Table 3. The punch load, LDH values and maximum strain prior to fracture of the blank all increased with increasing sheet thickness, which is not intuitive based on a simple analysis of material ductility. Previous reports from Tisza et al. [28] demonstrated that the forming limit of steel sheets increased non-linearly with an increase in sheet thickness, although such increases were far less than those induced by changes in material properties. The data summarized in Table 3 show that an increase of more than 81% in the punch load only yielded an increase of 21.4% in LDH and 16.8% in maximum strain prior to fracture. These increases, while not linearly related, appear to follow the same curve as shown in Figure 11.

## 4. Conclusions

An evaluation of weld metal formability of aluminum TWBs produced via FSW was made using an integrated DIC method in combination with an LDH press. The strain and displacement distribution and evolution in the LDH forming of AA5754 TWBs at various thicknesses ranging from 2.2 mm thick to 3.0mm thick were presented. The influence of the thickness of the base materials on the punch loading and formability was investigated.

The results of the present paper are summarized as follows:

(1) The formability of aluminum TWBs during LDH testing can be accurately determined with the assistance of digital image correlation techniques and the software ARAMIS.
(2) The distribution of strain of AA5754 TWBs produced via FSW in LDH testing is symmetrical along the section perpendicular to the weld seam when the thickness of both sides of the base material are the same, regardless of the thickness of the sheet. As such, no asymmetry associated with welding was found during the evaluation. Furthermore, as long as the sheet thickness on each side of the weld was similar, no asymmetry in strain distribution was reported.
(3) Increasing the sheet thickness of the aluminum TWB led to a commensurate increase in the LDH value, the strain at fracture and the load at fracture. These increases were not all linearly related with the increase in sheet thickness of the material.
(4) Increases in LDH values of aluminum TWBs are approximately linearly related to the increase in sheet thickness with a coefficient of determination (R^2^ value) of 0.94.

## Figures and Tables

**Table 1:** Chemical composition of AA5754.

Al	Si	Mn	Mg	Fe
Bal.	0.4	0.5	2.6–3.2	0.4

**Table 2:** Mechanical properties of AA5754.

Tensile Strength, MPa	Yield Strength, MPa	Elongation, %	Vickers Hardness
215	140	20	67.2

**Table 3:** Measured formability, punch loading, maximum strain and frame in the LDH test.

Thickness (mm)	Punch Loading (kN)	LDH (mm)	Maximum Strain	Stage
2.2	65.285	24.26	41.5	78
2.7	88.777	26.44	46.2	80
3.0	118.375	29.50	48.5	82

## Data Availability

Not applicable.
